# Wearable Safeguarding Leather Composite with Excellent Sensing, Thermal Management, and Electromagnetic Interference Shielding

**DOI:** 10.1002/advs.202302412

**Published:** 2023-07-09

**Authors:** Ziyang Fan, Liang Lu, Min Sang, Jianpeng Wu, Xinyi Wang, Feng Xu, Xinglong Gong, Tianzhi Luo, Ken Cham‐Fai Leung, Shouhu Xuan

**Affiliations:** ^1^ CAS Key Laboratory of Mechanical Behavior and Design of Materials Department of Modern Mechanics University of Science and Technology of China (USTC) Hefei 230027 China; ^2^ The First Affiliated Hospital of USTC Division of Life Sciences and Medicine University of Science and Technology of China Hefei Anhui 230036 P. R. China; ^3^ State Key Laboratory of Environmental and Biological Analysis Department of Chemistry The Hong Kong Baptist University Kowloon Hong Kong SAR 999077 P. R. China

**Keywords:** anti‐impact, EMI shielding, piezoresistive sensing, shear stiffening, smart electronic devices, thermal management

## Abstract

This work illustrates a “soft‐toughness” coupling design method to integrate the shear stiffening gel (SSG), natural leather, and nonwoven fabrics (NWF) for preparing leather/MXene/SSG/NWF (LMSN) composite with high anti‐impact protecting, piezoresistive sensing, electromagnetic interference (EMI) shielding, and human thermal management performance. Owing to the porous fiber structure of the leather, the MXene nanosheets can penetrate leather to construct a stable 3D conductive network; thus both the LM and LMSN composites exhibit superior conductivity, high Joule heating temperature, and an efficient EMI shielding effectiveness. Due to the excellent energy absorption of the SSG, the LMSN composites possess a huge force‐buffering (about 65.5%), superior energy dissipation (above 50%), and a high limit penetration velocity of 91 m s^−1^, showing extraordinary anti‐impact performance. Interestingly, LMSN composites possess an unconventional opposite sensing behavior to piezoresistive sensing (resistance reduction) and impact stimulation (resistance growing), thus they can distinguish the low and high energy stimulus. Ultimately, a soft protective vest with thermal management and impact monitoring performance is further fabricated, and it shows a typical wireless impact‐sensing performance. This method is expected to have broad application potential in the next‐generation wearable electronic devices for human safeguarding.

## Graphical abstract

Multifunctional wearable leather/MXene/shear stiffening gel (SSG)/nonwoven fabric (NWF) (LMSN) composites with integrated properties of electromagnetic interference shielding, piezoresistive sensing, thermal management, and safeguarding are fabricated via assembling the LM, SSG, and NWF. A novel intelligent protective vest is further developed based on LMSN composites, and it possesses high promise for next‐generation wearable electronic devices with human safeguarding.

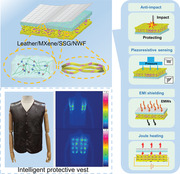

## 1 Introduction

As people's expectations of electronic devices become higher and higher, wearable technology has been developed into one of the largest growth industries. Different from traditional electronic devices, wearable ones possess ideal flexibility and convenience, and easily combine with the human body.^[^ ^1^, ^2^, ^3^, ^4^ ^]^ Recently, due to the surge of research on conductive polymers and flexible sensors,^[^ ^5^, ^6^, ^7^ ^]^ multifunctional wearable electronic devices have shown promising prospects in sensing, actuation, electronic skin, human health detection, and human–machine interaction.^[^ ^8^, ^9^, ^10^, ^11^, ^12^, ^13^, ^14^ ^]^ Sensors with excellent flexibility were crucial perception units in smart electronic devices and most of the previous works were focused on the high sensitivity and rapid response to external stimuli.^[^ ^15^, ^16^ ^]^ However, the stability and sensitivity under high energy impact which is very important for the practical application of electronic devices are often neglected due to the difficulty of structure designation. There is an urgent demand for wearable devices that can cope with the ubiquitous mechanical collision in daily life and high‐speed impacts in extreme situations, while sensing the impact simultaneously. Therefore, developing a facile, low‐cost, and efficient strategy for fabricating multifunctional wearable electronic devices integrated with high flexibility, good electrical conductivity, and superior impact resistance is highly required.

For thousands of years, leather has been a popular clothing material because of its good flexibility, high permeability, biocompatibility, as well as mechanical strength.^[^ ^17^, ^18^ ^]^ As an ancient natural material, leather with a 3D porous structure is composed of multilevel collagen fiber bundles, which enable itself to be a versatile matrix for flexible electronic devices.^[^ ^19^ ^]^ Ma et al. reported a multifunctional nanocomposite by decorating silver nanowires into leather via the facile vacuum‐assisted filtration process. The AgNWs/leather nanocomposites exhibited excellent Joule heating, electromagnetic interference shielding, and piezoresistive sensing behavior, which demonstrated high promise for next‐generation wearable electronic devices.^[^ ^20^ ^]^ Wang et al. directly edited the natural leather structure for obtaining multifunctional thermal camouflage armor by in situ growth of SiO_2_ nanoparticles.^[^ ^21^ ^]^ The special porous fiber structure and rough surface also endow the leather with good hydrophobicity and mechanical properties.^[^ ^22^ ^]^ Therefore, leather is considered a prospective substrate candidate in wearable electronic devices. However, although leather often has been used as a natural protective device since ancient times,^[^ ^23^ ^]^ the further improvement in its protection performance is still scarce. Moreover, it has been proved that the microstructure of the flexible substrates could significantly improve the sensing property of the flexible sensor.^[^ ^24^ ^]^ Regarding excellent flexibility and wearability, the integration of leather with versatile multifunctions plays a key role in developing high‐performance wearable electronic leathers with wonderful protecting behavior.

Shear stiffening gel (SSG), as a typical rate‐dependent material, has an obvious modulus enhancement effect under external stimulation,^[^ ^25^ ^]^ showing strong energy dissipation ability owing to phase transition (viscous state to solid state) during the impact process. Mostly, SSG had been used to strengthen flexible materials such as Kevlar fiber and polyurethane sponges, achieving good impact resistance performance.^[^ ^26^, ^27^ ^]^ Liu et al. embedded the SSG in a chitosan/MXene lamellar architecture skeleton, and the resulting composite effectively attenuated the impact force of 85.9−92.8%.^[^ ^28^ ^]^ Recently, a new type of negative Poisson's ratio skeleton composites filled with SSG has been developed and the coupling effect between the auxetic effect of the structure and the energy dissipation of SSG was clearly observed.^[^ ^29^ ^]^ The results showed that the appropriate structure could enhance the impact resistance of composites, and the laminated structure showed better impact resistance ability when impact velocity exceeded a critical range.^[^ ^30^ ^]^ Our previous work reported a toughness–flexibility coupling structure based on leather and conductive carbon nanotube (CNT)/SSG, which showed efficient anti‐impact and superior impact sensing performance.^[^ ^31^ ^]^ However, due to the low conductivity and weak shear thickening behavior of CNT/SSG nanocomposite, further improvement of the energy dissipation, sensitivity, electromagnetic interference (EMI) shielding, and thermal management for the leather composites remains an urgent challenge to be solved.^[^ ^32^, ^33^ ^]^

MXene, a 2D transition metal carbide, possesses outstanding metal‐like conductivity, large specific surface area, and excellent mechanical properties, shows extensive application potential in the thermal management and electromagnetic shielding fields. By a vacuum‐assisted filtration method, Gong et al. fabricated a phase change capsule/MXene/polyvinyl alcohol composite film based on MXene mortar, which realized Joule heating under the stimulation of low voltage (1.5 V) and excellent EMI shielding effectiveness values (43.13 dB).^[^ ^34^ ^]^ Xie et al. reported a high‐performance flexible piezoresistive sensor based on a 3D MXene/polyethylenimine network, which could monitor various human activities in real‐time and exhibit an excellent Joule heating performance.^[^ ^35^ ^]^ Obviously, owing to its superior conductivity and unique multisurface characteristics, the MXene is suitable for various substrate polymer materials to improve electric characteristics.^[^ ^36^ ^]^ In considering the porous fiber structure/rough surface, flexible/toughness nature, and sustainable natural resources, the MXene/leather composites possess large superiority in wearable electronics. Unfortunately, the relatively safe, comfortable, and multifunctional wearable electronic leather composite has not been well addressed.

Herein, this work reported a multifunctional leather/MXene/SSG/NWF (LMSN) composite with laminated structure prepared via suction filtration and assembling process, integrating capabilities of piezoresistive sensing, EMI shielding, anti‐impact, and thermal management together. The flexible and biocompatible leather was chosen as the substrate to carry MXene nanosheets. MXene nanosheets penetrated into the multilevel fiber structure of the leather to construct an efficient conductive network, which endowed the LM composites with excellent electrical performance. Then, the laminated structure of LMSN was assembled by introducing rate‐dependent SSG and nonwoven fabric (NWF) layers, and the product finally exhibited prime anti‐impact property of effectively buffering the impact force (65.5%) and high limit penetration velocity (91 m s^−1^). Interestingly, the LMSN composites could distinguish the low and high energy stimulus by exporting negative and positive resistance change, respectively. Finally, based on the synergy of each layer, an electrothermal protective vest for the human body was designed, realizing sensitive thermal management and impact monitoring by building the LM array area. As a result, the LMSN composites provided multiple protection for physical impact, low‐temperature injury, and electromagnetic radiation, demonstrating broad application prospects in wearable protective devices.

## 2 Results and Discussion

### 2.1 Preparation and Characterization of LMSN Composites

**Figure 1:**
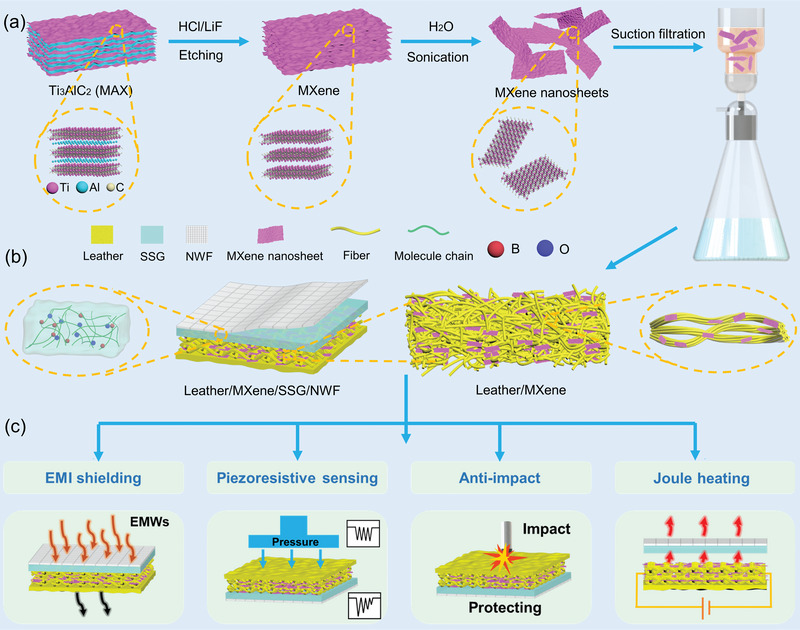
a) The fabrication process of the MXene nanosheets. b) The schematic diagrams show the preparation of the LMSN composite. c) LMSN composite with superb properties.

**Figure** **1**a shows the preparation process of the MXene nanosheets. First, the MAX phase powder was etched to form the multilayer MXene by hydrofluoric acid. Then, the reacted product was stripped by repeated ultrasonic and centrifugation in deionized water. Finally, the concentration was adjusted to obtain the required MXene nanosheets aqueous solution. Profiting from the hierarchical fiber structures of the natural leather, the LM composites were prepared by suction filtrating MXene aqueous solution. The MXene nanosheets were evenly distributed on the fiber network of leather, which could tightly attach to the collagen fiber bundles. Subsequently, the SSG layer and NWF layer were gradually assembled on the leather layer to form the laminated structure (Figure 1b). Because of the high affinity, the SSG could be easily combined with the rough fiber surface of the leather and the porous NWF. The SSG was a polypropylene siloxane colloid based on the B─O cross‐linking bond, prepared according to the method described above.^[^ ^31^ ^]^ At last, the LMSN composite with superb properties and diverse promising applications in wearable sensing, thermal management, and safeguarding was successfully prepared (Figure 1c).

**Figure 2:**
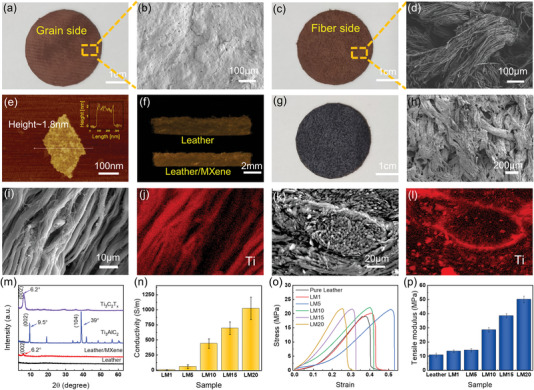
a) The digital and b) SEM images of the grain side of the leather. c) The digital and d) SEM images of the fiber side of the leather. e) The AFM image of the MXene nanosheet. f) The microscopic CT images of leather and LM. g) The digital and h) SEM images of the LM surface. i) The SEM and j) corresponding elemental mapping images of the LM surface. k) The cross‐sectional SEM and l) corresponding elemental mapping images of the LM. m) The XRD patterns of Ti_3_AlC_2_ precursor, Ti_3_AlC_2_, and LM. n) The electrical conductivity of LM composites with different MXene contents. o) The tensile mechanical property and p) the tensile modulus of LM composites.

**Figure** **2**a–d shows the digital and scanning electron microscope (SEM) images of leather with two different surfaces of grain side and fiber side. The grain side has a comfortable feel with a special granular structure, which is the upper layer of the leather. Figure 2b shows the SEM image of the uneven grains. The fiber side (Figure 2c) refers to the bottom layer of leather with plenty of natural collagen fibers formed in the leather‐making process. Figure 2d shows the SEM image of the fiber side, which clearly shows the closely interlaced 3D fiber network structure. In Figure S1a in the Supporting Information, the precursor Ti_3_AlC_2_ (MAX) phase shows the multilayer‐stacked structure. After etching and exfoliating, the MXene nanosheets show the nonordered thin‐flake structure (Figure S1b, Supporting Information). The transmission electron microscope (TEM) image captures the single MXene layer, which shows a transparent sheet‐like structure (Figure S1c, Supporting Information). The atomic force microscope (AFM) image of the MXene nanosheet shows a 2D lamellar structure with a thickness of 1.8 nm (Figure 2e), which further indicates the successful synthesis of the single‐layer MXene nanosheets aqueous solution.

According to literature reported,^[^ ^37^ ^]^ microscale pore structures can capture MXene lamella to prevent them from self‐stacking, advantaging the formation of an efficient conductive network. Therefore, the porous fiber network structure of the leather is suitable for combining with the MXene nanosheets. Then, the flexible LM composites were fabricated via vacuum suction filtration. The MXene nanosheets could easily penetrate into the multilevel fibers of leather under the pressure difference caused by filtration. Figure 2g shows the optical image of LM, the tawny fibers turn dark purple after the MXene nanosheets are attached to the fibers. The microscopic computed tomography (CT) image shows that the loose fiber structure of leather becomes tight (Figure 2f). After being wrapped by MXene nanosheets, the LM still maintains its unique 3D fiber structure (Figure S1d,e, Supporting Information). The SEM image of the LM fiber side also shows this point (Figure 2h). As can be seen, numerous fiber bundles penetrated by the MXene nanosheets tightly intertwine, each fiber bundle also includes many interlaced collagen fibers (Figure 2i). Then, the corresponding element mapping image of the Ti element indicates the successful penetration and uniform distribution of MXene nanosheets in every collagen fiber (Figure 2j). Further, the cross‐sectional microstructure of LM was characterized by the SEM. It could be seen that many fiber bundles were staggered and orderly in arrangement from the section (Figure S1f,g, Supporting Information). Figure 2k shows that each fiber bundle is composed of collagen fibers with almost the same direction, which are tightly woven to form the hierarchical structure to endow leather with a certain mechanical strength. Figure 2l shows the energy‐dispersive spectroscopy (EDS) mapping image of fracture collagen fibers and the Ti element exists on the cross‐section fibers, which indicates the MXene is evenly distributed in the 3D fiber structure of the leather. To further confirm that MXene exists in leather, the X‐ray diffraction (XRD) measurements of different materials are analyzed in Figure 2m. Ti_3_AlC_2_ shows a strong peak (104) of the Al layer at 39°, which disappears in MXene owing to etching during the preparation. The typical peak (002) moves from 9.5° to a lower angle of 6.2°, which indicates the distance between the MXene nanosheet layers increases. Compared with leather, the representative peak (002) is found in LM at 6.2°, indicating the presence of MXene nanosheets in leather fibers.

Then, LM composites (LM1, LM5, LM10, LM15, and LM20) with different MXene contents were prepared (Figure S2, Supporting Information). Their electrical conductivity is studied in Figure 2n. Pure leather is an insulating material, while the LM composite has excellent conductivity due to the penetration of MXene nanosheets. The electrical conductivity of LM1, LM5, LM10, LM15, and LM20 can reach up to 1.6, 56.5, 439.5, 693.8, and 1023.1 S m^−1^, respectively. With the increase of MXene content, the electrical conductivity of LM increases exponentially. Next, the tensile mechanical property of them is investigated in Figure 2o. The tensile stress of the different LM composites increases a little in comparison with pure leather. The fracture strain of the pure leather is 0.38, and LM5 reaches a maximum fracture strain of 0.51 with the increase of MXene content. It indicates that a certain amount of MXene can increase the fracture strain. Then the fracture strain gradually decreases to less than that of pure leather, which is 0.30 and 0.26 for LM15 and LM20, respectively. This phenomenon may be due to brittle fracture caused by stress concentration formed by excessive MXene accumulation. Nevertheless, the fracture strain of LM will still meet the demands of wearable devices. It is worth mentioning that the tensile modulus of all LM composites is higher than that of pure leather (Figure 2p), which is considered to strengthen the intertwist and friction between the fiber bundles due to attaching to collagen fibers through MXene.

**Figure 3:**
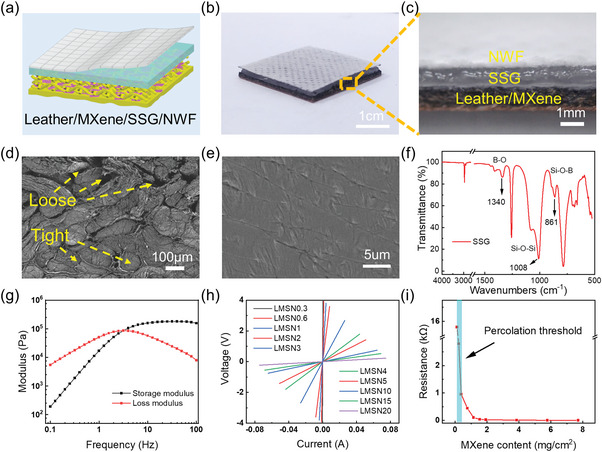
a) The schematic and b) optical images of LMSN. c) The cross‐sectional optical image of the LMSN. d) The cross‐sectional SEM image of the LM. e) The SEM image of SSG. f) The Fourier‐transform infrared (FT‐IR) spectrum analyses of SSG. g) The rheological properties of SSG. h) The conductivity of LMSN composites with different MXene contents. i) The percolation threshold of LMSN composites.

The LMSN composite was prepared by assembling the SSG layer and NWF layer on the LM composite to form the laminated structure (**Figure** **3**a,b). Figure 3c shows the cross‐sectional optical image of the LMSN composite. From the cross‐sectional SEM image of LM, it can be seen that the fibers on the fiber side are loose and have large pores (Figure 3d). SSG will slightly penetrate into the rough fiber surface of leather, making the three‐layer structure tightly connected due to its cold‐flow characteristic. Figure 3e shows the smooth surface of SSG. As shown in Figure 3f, the characteristic absorbance bands of SSG on 1340, 1008, and 861 cm^−1^ correspond to B─O, Si─O─Si, and Si─O─B groups, respectively, which proves the successful preparation of SSG. The rheological performance of SSG is shown in Figure 3g. As a classical rate‐dependent material, the storage modulus of SSG gradually increases from 191 to 169 370 Pa with frequency from 0.1 to 100 Hz, presenting an obvious shear‐stiffening effect. Additionally, there is an intersection point between the storage modulus curve and the loss modulus curve at 3.16 Hz. SSG is in the viscous flow state when the shear frequency is less than 3.16 Hz because the storage modulus is lower than the loss modulus. After the frequency reaches 3.16 Hz, the SSG changes into a rubbery elastic state owning to the storage modulus exceeding the loss modulus. This phase transition characteristic causes the modulus enhancement effect of SSG.^[^ ^38^ ^]^ Considering that SSG and NWFs were nonconductive, the copper electrode was adhered to the fiber side surface of the LM, and the electrical performance of the LMSN composite is measured in Figure 3h. Showing the *V*–*I* curves of LMSN composites with different MXene contents, the slope of the curves means the resistance of the samples is gradually decreasing. The percolation threshold of LMSN composites is pointed out in Figure 3i. When the MXene area fraction is 0.23 mg cm^−2^, the resistance reaches the kilo‐ohm level. With the MXene area fraction reaching 1.15 mg cm^−2^, the resistance falls below the hundred‐ohm level. The higher MXene area fraction results in a lower LMSN composite resistance.

### 2.2 EMI Shielding Performance of LMSN Composites

With the continuous advancement of flexible wearable electronic devices, more and more devices are used to prevent electromagnetic wave (EMW) pollution and shield humans from radiation. In general, the key factors affecting the electromagnetic shielding effect are the structure and conductivity of the materials. In view of the excellent conductivity of LM composites, they are considered promising candidate materials for electromagnetic shielding. Following the Schelkunoffs theory,^[^ ^39^ ^]^ the EMI shielding effectiveness (SE_T_) was used to evaluate the ability to shield EMWs with the calculation method in Discussion S1 in the Supporting Information.

**Figure 4:**
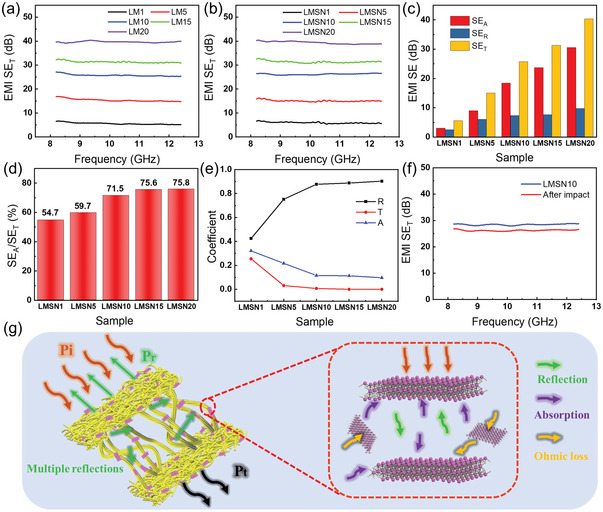
The EMI shielding effectiveness of a) LM and b) LMSN composites with different MXene contents. c) The contribution of reflection (SE_R_) and absorption (SE_A_) of LMSN composites. d) The percentage of SE_A_ to SE_T_ of LMSN composites. e) The *R*, *T*, and *A* coefficients of LMSN composites. f) The EMI shielding effectiveness of LMSN10 before and after impact. g) The schematic illustration of the EMI shielding mechanism.

As shown in **Figure** **4**a,b, because SSG and NWFs are nonconductive, the electromagnetic shielding properties (value of EMI SE_T_) of LM and LMSN are identical in the frequency range of 8.2–12.4 GHz (X‐band). The EMI SE_T_ of them is almost the same in the 8.2–12.4 GHz (X‐band), which are basically unaffected by frequency. Within the measurement range, the EMI SE_T_ of the sample is proportional to the electrical conductivity. The EMI SE_T_ of LMSN1, LMSN5, LMSN10, LMSN15, and LMSN20 are about 6, 15, 26, 31, and 40 dB, respectively. Note that the target value of the commercial EMI shielding materials is 20 dB, which can shield about 99% of incident EMWs energy in daily life. Therefore, LMSN10, LMSN15, and LMSN20 have reached the commercial standard, which can be used in promising EMI shielding applications. Figure 4c shows the contribution of reflection (SE_R_) and absorption (SE_A_) to the overall EMI SE_T_. It is obvious that SE_A_ of all samples accounts for a large proportion of SE_T_. As shown in Figure 4d, the percentages of SE_A_ in SE_T_ of LMSN10, LMSN15, and LMSN20 are as high as 71.5%, 75.6%, and 75.8%, respectively. So the LMSN composites focus on microwave absorption to shield EMWs. The *R*, *T*, and *A* power coefficients are calculated and used to support this point in Figure 4e. The *R* values of all samples exceed *T* and *A*, indicating that the EMW is mainly absorbed by multiple reflections inside the material. *R*‐value gradually increases with augmented MXene content, and the maximum value of *R* reaches 0.903. Furthermore, all the *T*‐values of the sample are very low. The LMSN10, LMSN15, and LMSN20 can block about 99% of EMWs, indicating that the LMSN composites possess high antipermeability performance to EMWs. The electromagnetic shielding effect of the LMSN10 composite after being hit by a hammer was measured (Figure 4f). The EMI SE_T_ value just decreases about 2 dB, which will hardly affect the effect of electromagnetic shielding, showing a high stability.

The outstanding EMI shielding performance of LMSN composites benefited from the excellent electrical conductivity and unique multilevel fiber structure of LM composites. Figure 4g shows the schematic diagram of the EMW shielding mechanism for LM composites. On the one hand, due to the high conductivity brought by MXene nanosheets, there is an interface impedance mismatch between the LM composites and the air. When EMWs touch this interface, part of that is reflected. After the incoming EMWs hit the MXene nanosheets on the leather fiber, they will interact with a large number of free electrons, resulting in ohmic loss and EMW energy absorption. On the other hand, the interlaced multilevel fiber structure of the LM composites causes the EMWs to occur in multiple reflections, and the EMWs will be repeatedly attenuated and absorbed by MXene nanosheets. In conclusion, LMSN composites display a superb EMI shielding effect. SSG and NWF of LMSN composite can further protect the interface of LM as the protective layers.

### 2.3 Piezoresistive Sensing Capability of LMSN Composites

**Figure 5:**
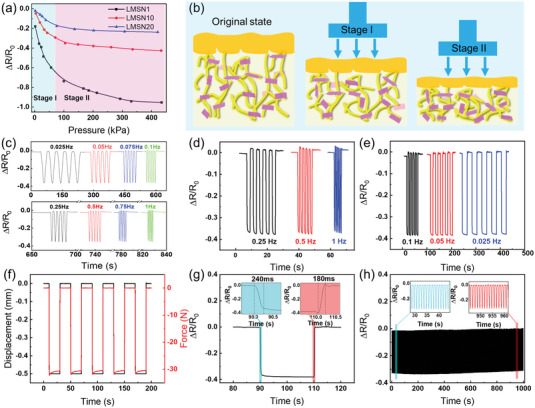
a) The relative resistance changes of LMSN composites upon various pressures. b) The schematic illustration of the piezoresistive sensing mechanism. c–e) The relative resistance changes of LMSN10 composite under the triangular and square waves with different frequencies. f) The force and displacement curve of the square wave at 0.025 Hz. g) The response time of the LMSN10 composite. h) The resistance changes of the LMSN sensor under 1000 cycles of loading/unloading.

LM composites possess excellent conductivity due to the generous MXene nanosheets attached to the leather fibers. Then, the copper electrode is adhered to the fiber side surface of the LM, and the SSG and NWF as the protective layer were assembled to form a LMSN piezoresistive sensor. **Figure** **5**a shows the piezoresistive sensing performances of the LMSN composites with different MXene contents under various external pressures. The relative resistance change (Δ*R*/*R*_0_ = (*R* − *R*_0_)/*R*_0_) is defined to characterize piezoresistive sensing ability, where *R*_0_ represents the initial resistance of the sample, *R* represents the resistance of the sample after applying pressure. The resistance changes of the LMSN composites increase as the pressure gradually increases, and possess a wide range of pressure perception (0–400 kPa), indicating their excellent piezoresistive sensing performance. During the entire compression process, the resistance gradually decreases, because the pressure causes the conductive layers in the 3D network to contact closely. According to the different applied pressures, the sensing ability of the LMSN sensors is divided into two stages. When the pressure is lower than about 70 kPa, the LMSN sensor shows a high sensitivity change (Stage I), which may be benefitted from the multilevel structure of the leather fiber being compressed to form a more compact 3D conductive network in Figure 5b. In the pressure range of 70–400 kPa (Stage II), as the leather fiber structure has been densified and the highly efficient conductive network has been constructed, the Δ*R*/*R*_0_ starts to decline slowly.

To further characterize the responsive behavior of the LMSN sensor, the Δ*R*/*R*_0_ of the LMSN10 sensor under excitation with different loading modes and different frequencies is tested. As illustrated in Figure 5c, the sensor exhibits almost the same Δ*R*/*R*_0_ under the excitation of the triangular waves with different frequencies when the pressure is kept constant. And it also can sense frequency changes, different frequencies have different response signals. Similar characteristics under square waves loading with different frequencies but different response signals are exhibited in Figure 5d,e. For example, during five times of square wave cyclic loading at 0.025 Hz (Figure S4a, Supporting Information), the changes of force and displacement with time are highly consistent with that of Δ*R*/*R*_0_ (Figure 5f). Therefore, the LMSN sensor is highly sensitive to detect the applied pressures, which possesses high precision and stable piezoresistive sensing performance. As shown in Figure 5g, a rapid response time of the LMSN sensor was measured, exhibiting different loading and unloading response times were 240 and 180 ms, respectively. Mechanical stability is an indispensable part of a piezoresistive sensor. Figure 5h presents the resistance change of the LMSN sensor under 1000 cycles of loading/unloading and the results indicate that the Δ*R*/*R*_0_ is always almost in a stable state, which proves that it has outstanding durability. In general, LMSN composites showed excellent piezoresistive sensing capability and mechanical stability, possessing extensive application potential for flexible wearable sensor fields.

### 2.4 Anti‐Impact Property of LMSN Composites

**Figure 6:**
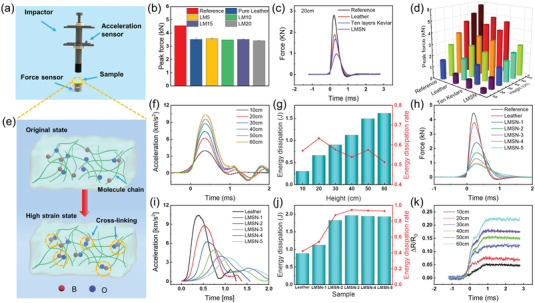
a) The schematic diagram of the drop hammer testing system. b) The peak forces of leather and LM composites at 40 cm. c) The force–time curves of diverse samples at 20 cm. d) The peak forces of diverse samples at the heights of 10–60 cm. e) The anti‐impact mechanism schematic diagram of SSG. f) The acceleration–time curves and g) energy dissipation of the LMSN composite at the heights of 10–60 cm. h) The force–time curves, i) acceleration–time curves, and j) energy dissipation of the LMSN composite with the different mass of SSG at 40 cm. k) The resistance changes of the LMSN composite under different impacts.

Attributed to the rate‐dependent characteristic of flexible SSG and high mechanical strength of toughness leather, the laminated LMSN composite is expected to become a potential impact‐resistant material. Therefore, the drop hammer impact test was used to investigate the anti‐impact property of LMSN composites. As shown in **Figure** **6**a, the sample was positioned in the center of a force sensor, and the impactor with an acceleration sensor was released to impact the sample under different heights. The force and acceleration data were acquired by an oscilloscope during the impact process. Then, the relationship between impact force and buffer time of LM composites was first tested under the drop hammer height of 40 cm (Figure S5a, Supporting Information). The peak force data of LM composites were compared, Figure 6b shows that LM5, LM10, LM15, and LM20 had almost the same peak force as pure leather and were lower than the reference value (reference means a control group of the impactor direct impacting the force sensor), which indicated the penetration of MXene sheets had little effect on the force‐buffering performance of the leather structure. Further, the anti‐impact property of the LMSN composite (LMSN10) was studied under the impactor release height at 10–60 cm (Figure S5b–g, Supporting Information). Pure leather and ten layers‐Kevlar (the mass is same as the LMSN) as the control group samples, the anti‐impact properties of the samples at the height of 10–60 cm have similar characteristics. For example, under the drop hammer height of 20 cm (Figure 6c), the LMSN possesses the lowest peak force and the longest force‐buffer time than other samples. Compared with the reference (2.78 kN), the peak force of LMSN (0.96 kN) is reduced by 65.5%. As shown in Figure 6d, the peak force data at different heights were summarized to compare. When the impact height was from 10 to 60 cm, the peak force of reference rapidly increased from 1.67 to 5.82 kN. The force‐buffering effect of the LMSN is far better than the other two samples, peak force only increases from 0.36 to 2.74 kN. The force attenuation efficiency of the LMSN has been maintained at more than half, this is because of the introduction of SSG. The anti‐impact mechanism diagram of SSG is shown in Figure 6e. The SSG was composed of polydimethylsiloxane molecular chains, in which the intermolecular cross‐linking between the polymer chains was usually through the p orbital of the B atom and the electron of the O atom to form the B─O bond. Due to the B─O crossing‐linking bonds are dynamic and transient, they would easily apart under the low strain rate stimuli (Figure S5h, Supporting Information). Nevertheless, when facing the stimulation of a high strain rate, B─O bonds could continue cross‐linking to block the movement of the molecular chain, which induced the SSG to transform into a hardening state (showing a rapid increase in modulus). Therefore, under the impact of the drop hammer, the sample will occur instantaneous hardening to resist the impact. Moreover, the hardening state on the macrography will produce cracks and deformation to dissipate the impact energy after being impacted (Figure S6a, Supporting Information).

To further study the effect of dissipated energy of the LMSN composite, the acceleration data were calculated to quantify energy. Figure 6f shows the curves of acceleration versus impact time of the LMSN under 10–60 cm. It is noteworthy that the evaluation is based on the first force peak, the rear peak is caused by the rebound after impact. With the impact heights gradually increasing, the energy dissipated by the LMSN also increases in Figure 6g (the energy data are calculated with the calculation method in Discussion S2, Supporting Information). The energy dissipation rate reaches a maximum value of 0.64 at 20 cm and then decreases slightly because the initial impact energy also increases with impact height. However, the energy dissipation rate is still maintained above 0.5, showing the LMSN composite has outstanding energy dissipation performance. Then, the mass of SSG in the LMSN is increased to continue to explore the force‐buffering and energy dissipation performance at the impact height of 40 cm (the X of LMSN‐X means X parts SSG, per SSG the same mass as that in LMSN is 0.6 g). As the number of SSG parts increases, the peak force gradually decreases from 2.4 to 0.9 kN (Figure 6h). The quality of SSG is positively related to the force‐buffering performance within a certain range. As shown in Figure 6i, the force‐buffering time has also increased significantly, which is conducive to dissipating more impact energy. The energy dissipation rate of LMSN‐1 is 0.54, then that of LMSN‐2, LMSN‐3, LMSN‐4, and LMSN‐5 is all greater than 0.8, which indicates a significant improvement of anti‐impact due to the increase in the mass of SSG (Figure 6j). In light of the excellent piezoresistive sensing performance of LM composites, the resistance change of LMSN composite under drop hammer impact is investigated in Figure 6k. After being impacted, the resistance of LMSN rose rapidly within about 1 ms, showed the reverse resistance changes to piezoresistive sensing (resistance reduction), and then the damage caused by the impact was difficult to recover. However, the resistance of the LMSN can recover in a long time due to the cold‐flow behavior of SSG. It is evident that as the impact height rises, the Δ*R*/*R*_0_ grows progressively. Eventually, the results indicate that the LMSN composite has prime anti‐impact performance and unique sensing response (different from piezoresistive sensing), showing excellent application potential for the intelligent protective clothing field.

### 2.5 Ballistic Impact Performance and Wireless Transmission System based on LMSN Composites

**Figure 7:**
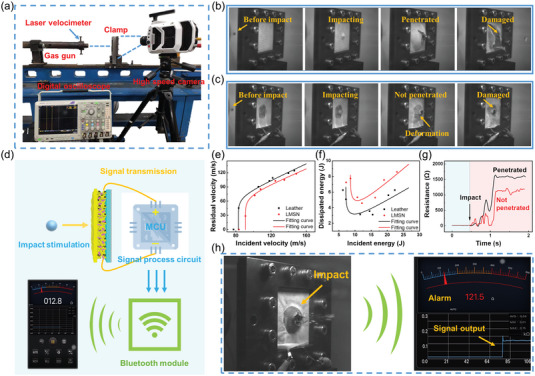
a) The diagram of the ballistic impact testing system. b,c) The high‐speed camera images of the impact process of leather and LMSN composite. d) The schematic diagram of the wireless sensing monitoring system. e) The residual velocity–incident velocity relationships of leather and LMSN composite. f) The energy dissipation results under different incident energy. g) The resistance changes of the LMSN composite at the penetrated and not penetrated states. h) The wireless alarm schematic diagram when the sample was penetrated.

To test the application potential of the LMSN composite in the field of protection, the ballistic impact performance of LMSN10 was tested by the ballistic system presented in **Figure** **7**a. The bullet is a steel ball with a diameter of 8 mm fired from the gas gun. The incident velocity (*v*_i_) is measured by a laser velocimeter with an oscilloscope and adjusted by controlling the pressure of the gas gun. The sample is fixed on the clamp and reinforced by bolts. The high‐speed camera is used to record the instant of sample destruction and calculate the residual velocity (*v*_r_) of the bullet.

The relationship between residual velocity and incident velocity is shown in Figure 7e. The curves were fitted according to the Recht–Ipson function^[^ ^40^ ^]^

(1) $$v_{r}=a\times {\left(v_{i}^{p}-v_{l}^{p}\right)}^{\frac{1}{p}}$$

where *v*_l_ is the limit penetration velocity of LMSN, *a* and *p* are the specific parameters. The function fitting curve has well coincided with the experimental point, indicating that the limit penetration velocity of LMSN is 91 m s^−1^, which is greater than 83 m s^−1^ that of leather. As shown in Figure 7b,c, under the impact of bullets at the same incident velocity of about 90 m s^−1^, pure leather will be penetrated directly. The LMSN composite can prevent the bullet from penetrating, showing better anti‐impact performance. When the *v*_i_ is greater than *v*_l_, the leather and the LMSN will be penetrated by bullets. Due to the modulus enhancement effect of SSG under external stimulation, the residual velocity of the LMSN is always less than that of leather. However, when the *v*_i_ is high enough, the gap between the residual velocity of leather and that of LMSN will gradually narrow because the shear stiffening property of SSG will be weakened with the increasing velocity. Furthermore, defining the dissipated energy as

(2) $$E_{d}=\frac{1}{2}mv_{i}^{2}-\frac{1}{2}mv_{r}^{2}$$

where *E*_d_ is the energy dissipation and *m* is the bullet mass. As illustrated in Figure 7f, when the samples are not penetrated, the impact energy of the bullet is completely dissipated. When the samples are penetrated, with the incident energy increasing, the dissipated energy decreases first and then increases. The energy dissipated by LMSN is always greater than that of leather, indicating better ballistic energy dissipation performance.

In addition, a voltage‐divided circuit was used to measure the resistance change of LMSN during high‐velocity ballistic impact (Figure 7g). After being impacted, the resistance rose rapidly after about 1 ms. The resistance response of the LMSN after penetration was greater than that of not penetrated, which was because the ballistic impact during penetration caused greater damage to the conductive network inside the LM. Here, a wireless impact sensing application was designed to catch this unique impact sensing feature of the LMSN composite. A wireless transmitting system was connected with the LMSN and signals could be received through a smartphone (Figure 7d). As displayed in Figure 7h, the safety resistance of the LMSN was set to be less than 30 Ω in the phone. Once the LMSN was damaged by impact, the resistance would rise rapidly to 121.5 Ω. The mobile phone would receive a resistance signal and then the system alarmed immediately (Videos S1 and S2, Supporting Information). Therefore, combined with the characteristics of wireless transmission, the LMSN composite has considerable development prospects in the field of intelligent wearable safeguarding.

### 2.6 Electrothermal Performance of LMSN Composites

**Figure 8:**
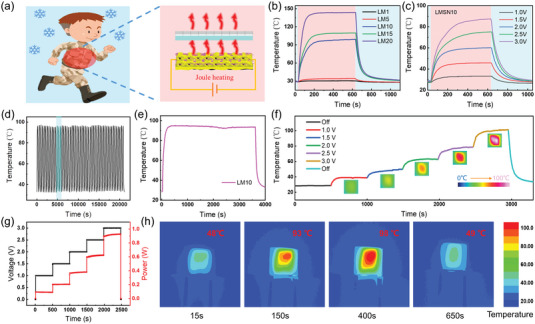
a) Schematic of LMSN‐based Joule heating effect (partial illustrations are sourced from the freepik website). b) The temperature–time curves of LM composites with different MXene contents at 3.0 V. c) The temperature–time curves of LMSN10 at different voltages. d) The temperature–time curves of LM10 under 50 heating/cooling cycles. e) Long‐term Joule heating performance of LM10 at 3.0 V. f) The step precision heating performance of LM10 at 1.0–3.0 V. g) The corresponding voltage and power changes curves of (f). h) The IR camera images of the Joule heating process at 3.0 V.

The LM composites with low resistance can work as wearable electrothermal devices based on the Joule heating effect (**Figure** **8**a). Figure 8b shows the time–temperature relationship of the LM with various MXene contents at the voltages of 3.0 V. When voltage is applied to both ends of the LM composites by the electrodes, they are heated rapidly due to the generated Joule heat and then reach a relatively stable saturation temperature. The saturation temperature of LM1, LM5, LM10, LM15, and LM20 is about 30, 34, 98, 109, and 143 °C, respectively. At the higher MXene content, the LM composite shows higher saturation temperature under the same voltage due to a more efficient 3D conductive structure. Noting that LM10 easily reaches saturation temperature (about 93 °C) in 150 s and possesses a stable platform period at saturation temperature, the corresponding heat generation effect is visually recorded by using an IR camera (Figure 8h). Since LM10 has achieved excellent electrothermal saturation temperature at 3.0 V, it is chosen for further electrothermal performance research. Figure S7a in the Supporting Information presents it under low supplied voltages from 1.0 to 3.0 V. The heat generation process is also divided into three stages: heating, steady‐state, and cooling. Significantly, the saturation temperature of the LM10 composite increases with the increasing supplied voltage, demonstrating its good control effect by voltage. The LMSN10 composite has the same excellent electrothermal performance in Figure 8c. The saturation temperature comparison between the LM10 and the LMSN10 is shown in Table S1 in the Supporting Information. Due to the weak heat insulation effect of SSG, the saturation temperature of the LMSN10 is reduced a little. Surprisingly, the saturation temperature of the LMSN10 still reaches 87 °C at 3 V, which is far higher than the temperature required for commercial electric heating clothing.

Furthermore, to explore the stability and durability of LM10 composite as a wearable electrothermal device, the cyclic test was conducted by heating/cooling at 3.0 V (Figure 8d). Figure S7b in the Supporting Information shows the enlarged curve of the light blue part in Figure 8d with three cycles. There is almost no difference in the temperature curve under 50 heating/cooling cycles, indicating the well electrothermal stability for the LM10 composite. Figure 8e shows the heating working curve of the LM10 composite within 1 hour, verifying its long‐term durability. Then, the temperature change was measured when the voltage is controlled from 1.0 to 3.0 V for a long time (Figure 8f). The saturation temperature of different voltages is still stable and well matching with the change time of voltage (Figure 8g). It shows that the electrothermal performance of the LM10 composite can be accurately adjusted by voltage and has excellent response performance. The power change is also nearly stable during the whole process, this characteristic indicates that it can be a reliable wearable electrothermal device that conforms to Joule's law. Therefore, the designed LMSN structure can be believed to have important applications in thermal management and safety protection of the human body.

### 2.7 Personal Safety Protection Vest with Thermal Management Performance

**Figure 9:**
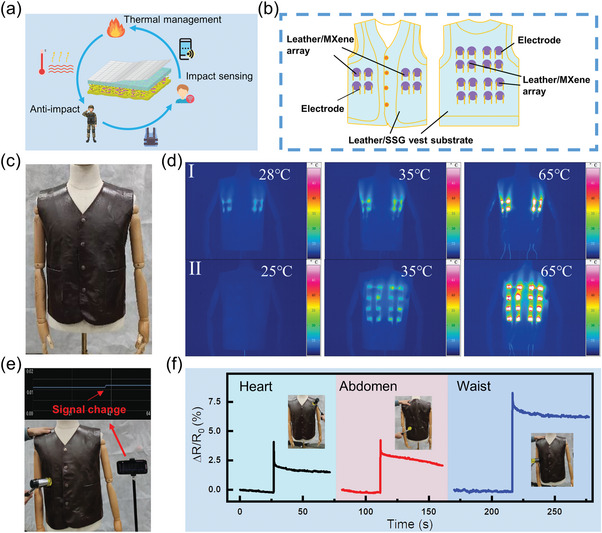
a) Practical applications of the LMSN composite (partial illustrations are sourced from the freepik website). b) The design diagram of the personal safety protection vest. c,d) The optical image and IR camera images of the vest on a mold. The resistance changes caused by knocking are monitored by e) a wireless transmission system and f) the impedance meter test system.

The soldiers on duty at the border are often in a harsh cold environment, it is sometimes difficult to take into account the effect of providing temperature for their bodies when wearing armor. So the demand for protective clothing with thermal management performance is increasingly urgent. The excellent anti‐impact and thermal management performance of LMSN composites prove their application potential in this field (**Figure** **9**a). Consequently, a 3 × 3 array was first integrated on a piece of leather, which could achieve regional heating (Figure S8a,b, Supporting Information). Then, the electric heating vest was designed based on a leather substrate in Figure 9b, the array design enabled the heat generation area to cover the human body.^[^ ^41^, ^42^ ^]^ With an LM10 as an array sub‐unit, two 2 × 2 LM arrays and one 4 × 4 LM array were designed on the front chest and back of the leather vest, respectively. Then, each LM10 sub‐unit was connected by an electrode and wire. Finally, like the structure of LMSN composites, the SSG and NWF were assembled on the leather vest substrate to complete the preparation of the electrothermal body safeguarding vest. As exhibited in Figure S8c in the Supporting Information, the vest was beautifully worn on the human body, indicated the wearable comfort and practicality of the electrothermal body safeguarding vest. Furthermore, the electrothermal performance of the leather vest was tested with a human mold (Figure 9c). As shown in Figure 9d, the IR images of the front (series I) and back (series II) of the leather vest under different voltages were taken by the infrared radiation (IR) camera. A total of 24 electric heating subunits form an array, which can cover the front chest and back to provide temperature for the main trunk of the human body. By adjusting the supply voltage, the temperature of the electrothermal vest can easily reach two levels, which are about 35 and 65 °C (the effective demand of common commercial heating clothes), respectively. Moreover, the security status of the array area can also be detected by connecting the leather vest to the Bluetooth transmission module. As exhibited in Figure 9e and Videos S3 and S4 in the Supporting Information, when the human body's key parts of the heart, abdomen, and waist were hit by a hammer, the mobile phone would simultaneously receive the resistance changes to achieve the effect of real‐time monitoring. The impact of a hammer was smaller, resulted in smaller resistance changes. The specific resistance response data were measured by the impedance meter in Figure 9f. The small resistance change (Δ*R*/*R*_0_ is about 5%) could also be monitored, which also verified the accuracy of the Bluetooth transmission module. To conclude, the electrothermal vest has both electric heating capacity and impact‐response characteristics, which will be expected to apply in protective clothing in cold environments.

## 3 Conclusion

In summary, this work reported an LMSN composite via assembling the LM, shear stiffening gel, and NWF. The MXene nanosheets were immersed in the porous fiber network structure of leather through suction filtration to form an efficient 3D conductive network, which endowed the LM with excellent conductivity. Therefore, the LM composites showed superior EMI shielding, piezoresistive sensing, and Joule heating properties. Under a low MXene content, the LM10 composite achieved an EMI SE_T_ of about 26 dB due to the ohmic loss and multiple reflections of EMWs in multistage fiber structure, a high saturation temperature of about 98 °C at 3.0 V and precise temperature control by voltage due to the resistance stability. Moreover, the LMSN composite also showed a high saturation temperature of about 87 °C at 3.0 V and identical EMI shielding capability after assembling SSG and NWF. Interestingly, the LMSN composites possessed better anti‐impact performance in comparison to the LM composites. On account of the rate‐dependent characteristic and energy dissipation performance of SSG, the LMSN composites possessed an obvious force‐buffering (about 65.5%) and energy dissipation (above 50%) performance, and a high limit penetration velocity of 91 m s^−1^, which were superior to the pure leather. Moreover, the LMSN composites could distinguish the low and high energy stimulus by exporting negative and positive resistance change, respectively. Eventually, a novel intelligent protective vest was designed and it showed excellent thermal management and wireless impact‐sensing performances, which demonstrated the application potential of the LMSN composites for next‐generation wearable electronic devices with human safeguarding.

## 4 Experimental Section

### Materials

Natural cow leather (≈1 mm in thickness) and NWF were the commercially available product, China. Ti_3_AlC_2_ MAX powder with 400 mesh used in this study was purchased from 11 Technology Co., Ltd., Jilin, China. Lithium fluoride (LiF) was purchased from Aladdin Chemical Co., Ltd., Shanghai, China. Hydrochloric acid (HCl), boric acid, hydroxyl silicone oil (500 mm^2^ s^−1^, AR degree), and octanoic acid were provided by Sinopharm Chemical Reagent Co. Ltd., Shanghai, China. All chemical reagents were used as received without further purification.

### Fabrication of SSG

SSG was prepared according to previous work.^[^ ^43^ ^]^ First, boric acid and silicone oil were mixed and stirred uniformly with a mass ratio of 1:20, followed by the mixture being heated in an oven at 180 °C for 2 h. Then the octanoic (250 µL per 200 g hydroxyl silicone oil) was added to the mixture, and continued to heat for 30 min in the oven. Finally, the SSG was obtained after cooling the mixture to room temperature.

### Preparation of MXene Aqueous Solution

The delaminated MXene nanosheets were synthesized according to the methods described previously.^[^ ^44^ ^]^ First, LiF (4 g) and HCl (80 mL) were mixed into the homogenous solution in a Teflon beaker, followed by adding Ti_3_AlC_2_ MAX powder (4 g) at a slow rate. Afterward, the mixture solution reacted for 24 h at 35 °C to etch the Al layers from the Ti_3_AlC_2_ phase. To obtain the multilayer MXene, the reacted product needed to be washed repeatedly with deionized water until the solution pH reached ≈6. Then, the above multilayer MXene was continued with ultrasonic treatment in ice water for 2 h and centrifuged at 3500 rpm for 1 h to exfoliate multilayer MXene. Finally, the MXene aqueous solution was obtained by collecting the exfoliated supernatant.

### Fabrication of LMSN Composites

The industrial substances of the leather surface were removed by alcohol processing. Subsequently, the leather was fixed on the suction filtration device with the fiber side on the top. The MXene nanosheets were uniformly dispersed into the multistage fiber network of the leather by vacuum filtrating for 2 h. Then, a series of flexible LM composites with different MXene mass fractions were fabricated by filtrating different volumes of MXene aqueous solution. The LM with MXene aqueous solution filtration volumes of 1.0, 5.0, 10, 15, and 20 mL were, respectively, named LM1, LM5, LM10, LM15, and LM20. Finally, the SSG layer and NWF layer were step‐by‐step assembled on LM composites to form the LMSN laminated structure.

### Characterization

The microstructure of materials was characterized by an SEM (Gemini SEM 500, ZEISS) equipped with an EDS system. The MXene nanosheets were observed by field‐emission TEM (FETEM, JEM‐2100F). The height morphology of MXene nanosheets was characterized by the AFM (dimension icon, Bruker). The crystalline phase of MAX powder, MXene, leather, and LM composite was characterized by XRD (SmartLab). The FT‐IR spectra were tested by a Nicolet Model 759 spectrometer. The EMI shielding performance of different samples in the 8–12 GHz region (X‐band) was explored by a commercial vector network analyzer (AV3672, China electronics technology instruments Co., Ltd., China) with a waveguide cavity.

### Mechanical Testing

The rheological properties of the SSG were investigated by a rheometer (Physica MCR 302, Anton Paar Co., Austria). A parallel plate with a diameter of 20 mm and a fixed gap of 1 mm was adopted. A certain mass of SSG was taken the oscillation frequency scanning with a shear frequency range of 1–100 Hz for testing. The mechanical performance of LM composites was tested by the material test system (MTS, ASTM D‐882). The electrical performance of the sample was measured by the ModuLab materials test system (Solartron Analytical, AMETEK Advanced Measurement Technology, Inc., UK). The anti‐impact properties tests were evaluated including the low‐velocity drop hammer impact test and the high‐velocity ballistic impact test. In the drop hammer test system (ZCJ1302‐A, MTS System Co., America), the hammerhead weighing 0.56 kg was released from 10 to 60 cm, and the sample was placed on the center of the force sensor. An oscilloscope was used to collect force and acceleration data. The ballistic impact test system was consisted of a high‐speed camera, a gas gun, a laser velocimeter, and a digital oscilloscope. The bullet used was a small steel ball with a mass of 2.08 g.

### Statistical Analysis

The quantitative data were presented as mean ± standard deviation (s. d.) from at least three measurements. Normalization treatment was performed on the resistance to characterize the sensing performance. Pre‐processing of data for (EMI) shielding and impact testing could be found in Discussion S1 and S2 in the Supporting Information.

## Conflict of Interest

The authors declare no conflict of interest.

## Supporting information

Supporting Information

Supplemental Video 1

Supplemental Video 2

Supplemental Video 3

Supplemental Video 4

## Data Availability

The data that support the findings of this study are available from the corresponding author upon reasonable request.
