# Supplementary material for: Wearable Safeguarding Leather Composite with Excellent Sensing, Thermal Management, and Electromagnetic Interference Shielding
Source: Adv Sci (Weinh). 2023 Jul 9;10(26):2302412. doi: 10.1002/advs.202302412 (PMC10502653; doi:10.1002/advs.202302412)
Supplement: Supplementary file 1 — Supporting Information [file ADVS-10-2302412-s002.pdf]

ADVANCED SCIENCE
Open Access

Supporting Information

for *Adv. Sci.*, DOI 10.1002/advs.202302412

Wearable Safeguarding Leather Composite with Excellent Sensing, Thermal Management, and Electromagnetic Interference Shielding

Ziyang Fan, Liang Lu, Min Sang, Jianpeng Wu, Xinyi Wang, Feng Xu, Xinglong Gong*, Tianzhi Luo, Ken Cham-Fai Leung and Shouhu Xuan**

Supporting Information

Wearable Safeguarding Leather Composite with Excellent Sensing, Thermal Management, and Electromagnetic Interference Shielding

Ziyang Fan[#], Liang Lu[#], Min Sang[*], Jianpeng Wu, Xinyi Wang, Feng Xu, Xinglong Gong[*], Tianzhi Luo, Ken Cham-Fai Leung, Shouhu Xuan[*]

Z. Fan, M. Sang, J. Wu, X. Wang, F. Xu, X. Gong, T. Luo, S. Xuan

CAS Key Laboratory of Mechanical Behavior and Design of Materials

Department of Modern Mechanics

University of Science and Technology of China (USTC)

Hefei 230027, China

E-mail: sm019050@ustc.edu.cn, gongxl@ustc.edu.cn, xuansh@ustc.edu.cn

L. Lu

The First Affiliated Hospital of USTC

Division of Life Sciences and Medicine

University of Science and Technology of China

Hefei, Anhui, 230036, PR China

K. Leung

State Key Laboratory of Environmental and Biological Analysis

Department of Chemistry

The Hong Kong Baptist University

Kowloon, Hong Kong SAR, PR China

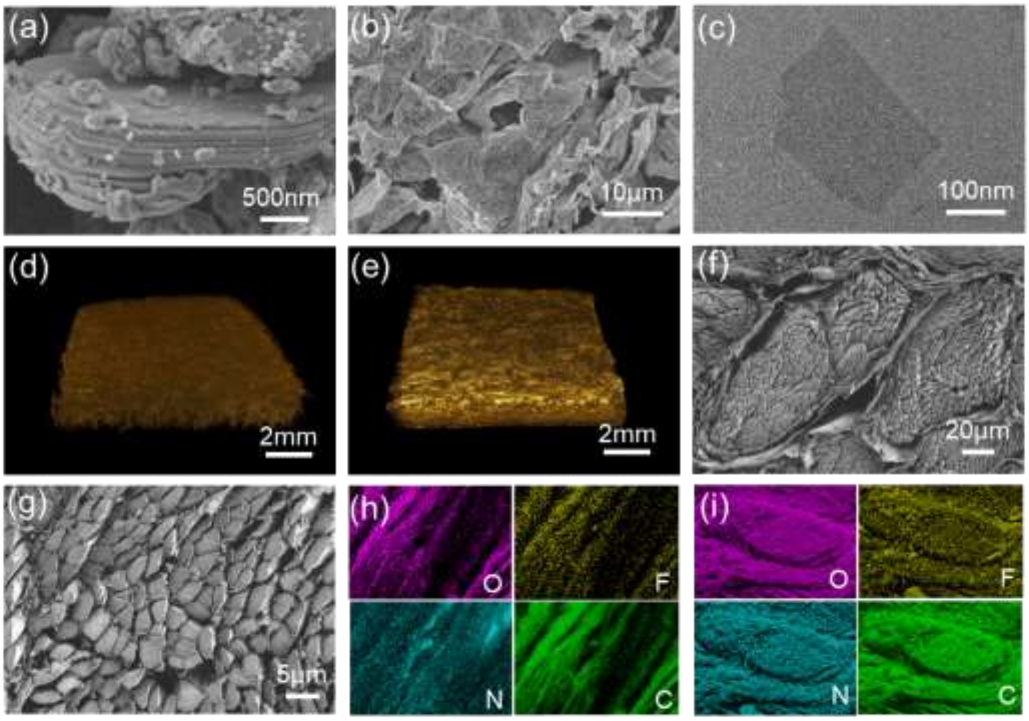

Figure S1. (a) The SEM image of Ti_3AlC_2 precursor. The SEM (b) and TEM (c) images of the MXene nanosheets. The microscopic CT images of leather (d) and Leather/MXene (e). (f-g) The cross-sectional SEM image of the Leather/MXene. The elemental mapping images of the surface (h) and cross-section (i) of the Leather/MXene.

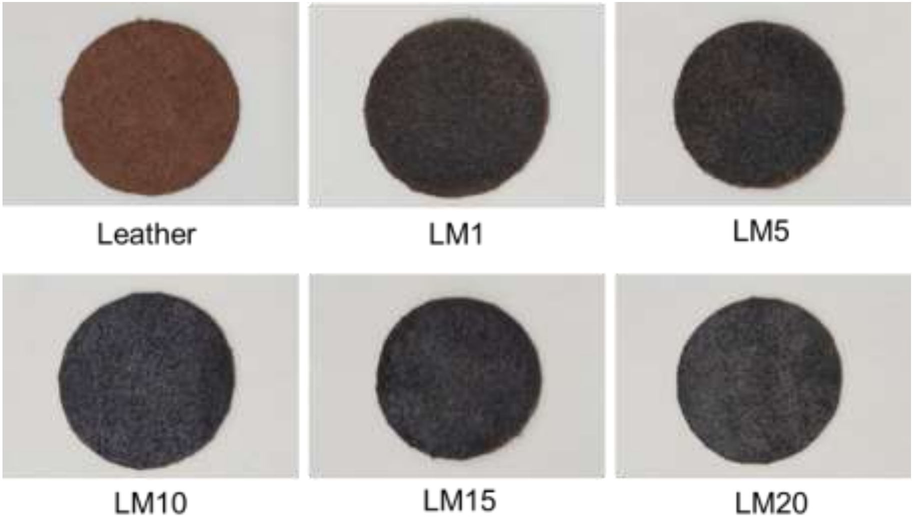

Figure S2. The optical images of Leather/MXene composites with different MXene contents.

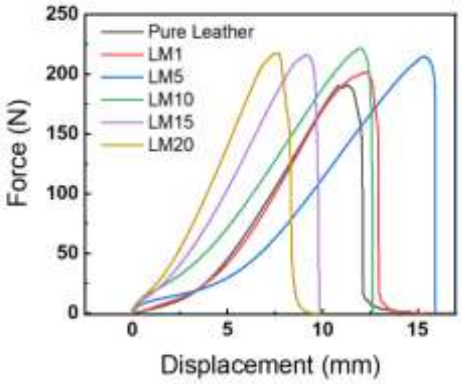

Figure S3. The force-displacement of Leather/MXene composites with different MXene contents.

Supplementary Discussion 1: The EMI shielding effectiveness (SE_T) includes reflection (SE_R), absorption (SE_A), and multiple reflections (SE_M), which is used to evaluate the performance to resist incident EMW. Because SE_M can be ignored when the SE_T value exceeds 15 dB, the calculation formula of relevant parameters in EMI SE_T are as follows:

$$SE_T = SE_R + SE_A \quad (1)$$

Where, SE_T, SE_R, and SE_A were calculated by the power coefficients of reflection (R), absorption (A), and transmission (T). R, A, and T were calculated from S_{11} and S_{21} parameters measured by the vector network analyzer.

$$\mathrm{R} = -10^{(S_{11}/10)},\ \mathrm{T} = 10^{(S_{21}/10)},\ \mathrm{A} = 1 - \mathrm{R} - \mathrm{T} \quad (2)$$

$$SE_R = -10\log(1 - R) \quad (3)$$

$$SE_A = -10\log(T/1 - R) \quad (4)$$

$$SE_T = -10\log T \quad (5)$$

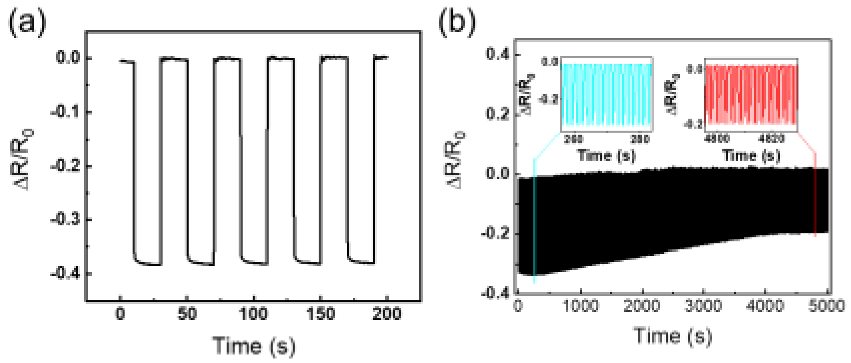

Figure S4. (a) The corresponding response signal of the square wave at 0.025 Hz. (b) The resistance changes of the LMSN sensor under 5000 cycles of loading/unloading.

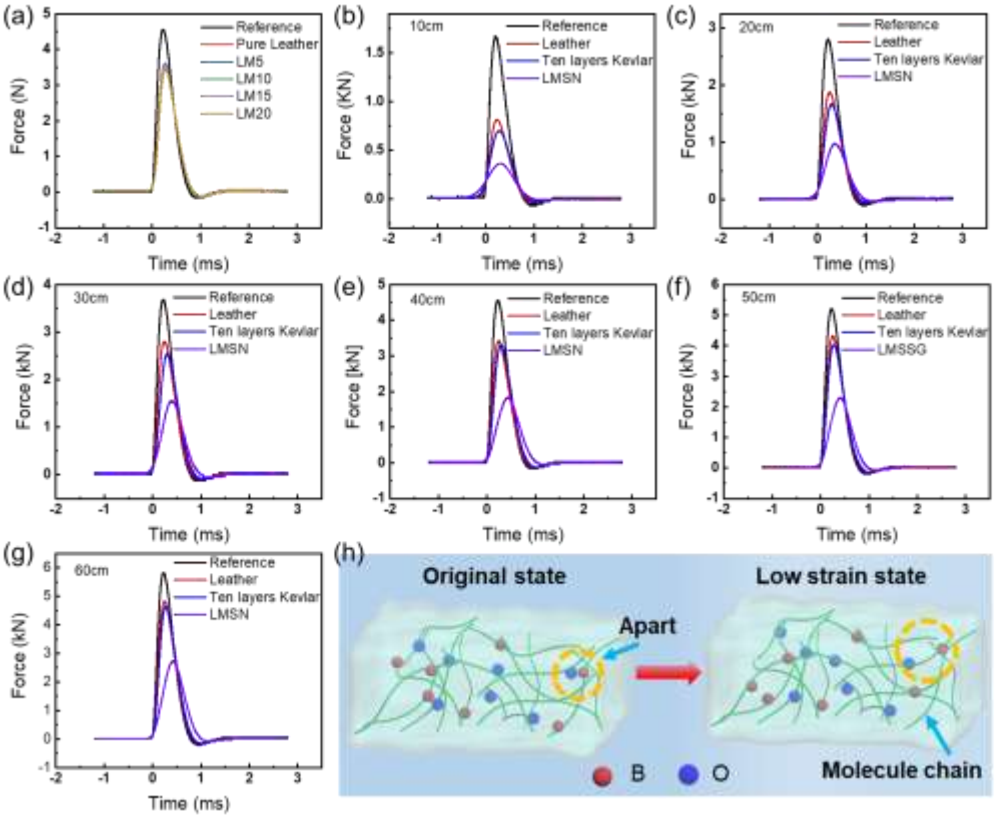

Figure S5. (a) The force-time curves of leather and LM composites at 40cm. (b-g) The force-time curves of diverse samples at 10-60 cm. (h) The mechanism schematic diagram of SSG at low strain rate state.

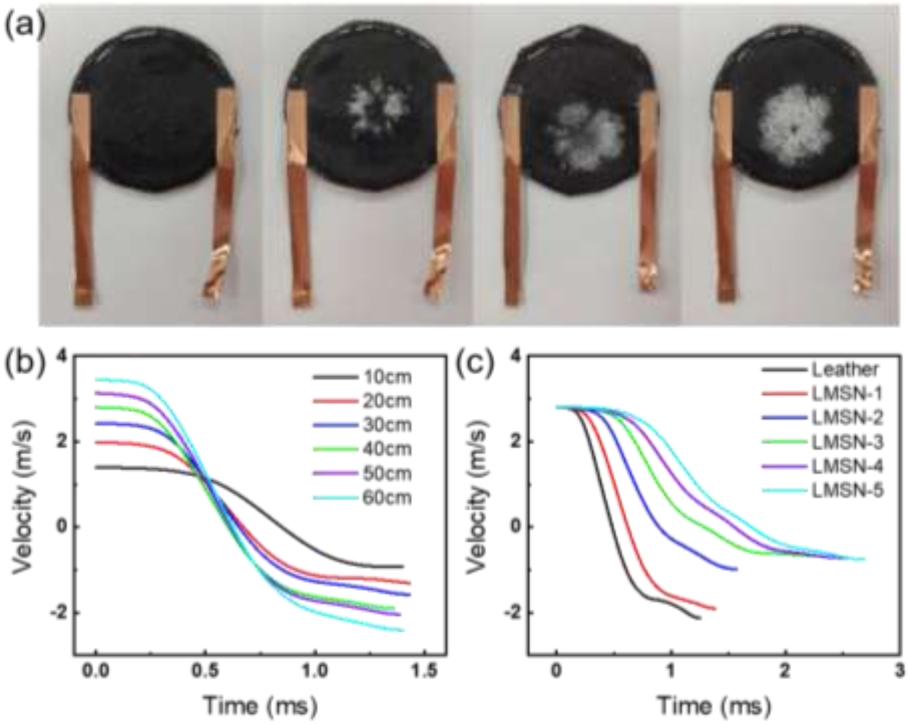

Figure S6. (a) The optical images of LMSN under different impacts. (b) The corresponding velocity-time curves of the LMSN composite at the heights of 10-60 cm. (c) The corresponding velocity-time curves of the LMSN composite with the different mass of SSG at 40 cm.

Supplementary Discussion 2: The acceleration changes of the sample during impact using an acceleration sensor to collect. Then, integrating the acceleration to obtain the velocity change by formula (7) and the dissipation of energy data is calculated using the kinetic energy theorem (8). The initial energy during the impact process is calculated by the formula (6). Finally, the energy dissipation rate is obtained by formula (9).

$$E_0 = \mathrm{mgh} = \frac{1}{2}mv_0^2 \tag{6}$$

$$v = v_0 + \int adt \tag{7}$$

$$E_d = \frac{1}{2}mv^2 - \frac{1}{2}mv_0^2 \tag{8}$$

$$E_{dr} = \frac{E_d}{E_0} \tag{9}$$

Where E_0 is the initial impact energy of different impact heights, v_0 is the initial impact velocity of different impact heights, a is the acceleration data of different impact heights, E_d is the energy dissipation of the sample during the impact process.

E_{dr} is the energy dissipation rate.

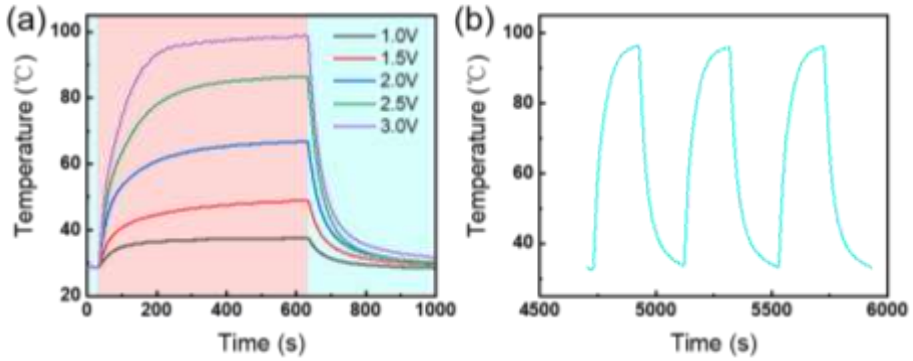

Figure S7. (a) The temperature-time curves of LN10 at different voltages. (b) The temperature-time curves of LM10 under 3 heating/cooling cycles.

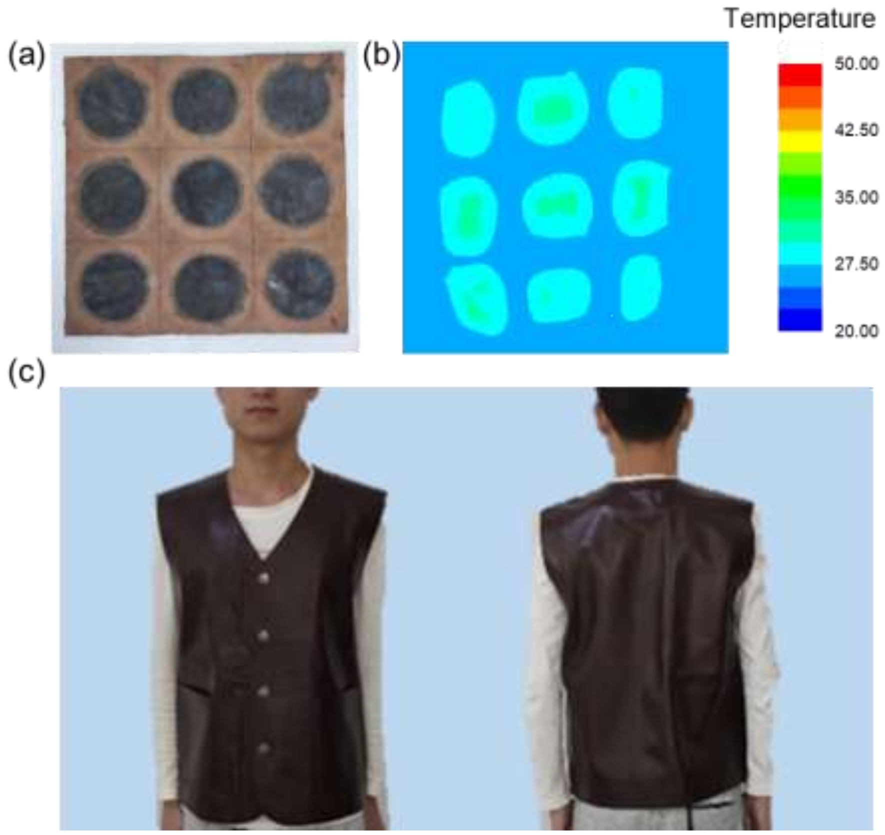

Figure S8. (a) the Leather/MXene with the 3 * 3 array. (b) The infrared camera image of the Joule heating effect. (c) the optical image of the personal safety protection vest on the human body.

Table S1. The saturation temperatures of LM and LMSN at different voltages

voltage	Leather/MXene	Leather/MXene/SSG/NWF
1.0 V	37℃	34℃
1.5 V	48℃	45℃
2.0 V	66℃	60℃
2.5 V	86℃	75℃
3.0 V	98℃	87℃
